# Evidence-based development of new qualification models for public health practice and science

**DOI:** 10.1093/eurpub/ckac131.383

**Published:** 2022-10-25

**Authors:** L Arnold, S Bimczok, N Dragano, S Götz, A Kietzmann, A Melville-Drewes, S Weyers, D Starke

**Affiliations:** 1 Applied Research and Transfer, Academy of Public Health Services, Duesseldorf, Germany; 2 Institute of Medical Sociology, Centre for Health and Society, University of Düsseldorf, Duesseldorf, Germany; 3 Health Department, State Capital Düsseldorf, Duesseldorf, Germany

## Abstract

The Public Health Service (PHS) needs a sufficient and well-trained workforce to fulfill core public health activities. Given the multitude of complex challenges, from often short-term crisis management to long-term health promotion, a better integration of science and practice in the PHS is essential. In Germany, there is a lack of systematically established cooperations and integrated training concepts. However, both are essential to ensure continuous knowledge transfer between science and practice. In the EvidenzÖGD study, representatives from practice, teaching and science jointly develop and pilot new qualification models to qualify public health professionals for work at the interface of practice and science. Based on a systematic analysis of existing collaborations and qualification models, 23 expert interviews were conducted to identify qualification approaches that will enable future public health professionals to work at the interface between science and practice. In a stakeholder workshop planned for July 2022, a multistage decision-making process based on strategic orientation mapping will be conducted to prioritize the identified approaches and generate ideas for new qualification models. In total 17 papers were analyzed and inductively coded according to three main categories. In addition to (1) context-related aspects, such as credentialing, recruitment strategies and resources, also (2) content-related aspects, such as training forms and didactical concepts were extracted. Furthermore, criteria relevant for the further development and continuation of the models (3) could be identified. By strengthening the scientific nature of practice in PHS and anchoring PHS-relevant topics in academia, the EvidenzÖGD project contributes to a sustainable strengthening of evidence-informed approaches in PHS practice. The evidence-informed, participatory development of a tailored qualification model is intended to promote the pilot of the model, which is due in 2023.

**Key messages:**

• Integrated qualification models support the strengthening of evidence-based approaches in the public health service (ÖGD) and promote the integration of ÖGD-relevant issues in science.

• Participatory approaches with explicit consideration of the perspective of young health professionals ensures that new qualification approaches meet the needs and requirements of future generations.

